# A Case of Takotsubo Syndrome in a Smoker-Epileptic Patient With Rhinovirus Pneumonia in the Intensive Care Unit: Could the InterTAK Criteria Be Useful?

**DOI:** 10.7759/cureus.69638

**Published:** 2024-09-18

**Authors:** Simona Tantillo, Martina Guarnera, Francesco Benvenuti, Irene Ottaviani, Nicola Cilloni

**Affiliations:** 1 Department of Anesthesia, Intensive Care and Prehospital Emergency, Maggiore Hospital Carlo Alberto Pizzardi, Bologna, ITA

**Keywords:** acute hypoxemic respiratory failure, critically ill patients, pneumonia complication, respiratory disease, takotsubo syndrome

## Abstract

Takotsubo syndrome (TS) is an acute cardiac dysfunction that typically presents hypokinesis of the apical segment of the left ventricle beyond a single coronary artery territory. The pathological mechanisms of TS remain unclear, and several possible theories have been postulated, including catecholamine excess, coronary artery spasm, microvascular dysfunction, and metabolic disturbances. Based on the etiology, a primary and secondary form is distinguished. In primary TS, acute cardiac symptoms are the primary reason for seeking acute medical care. In secondary TS, the syndrome occurs in patients already hospitalized for a medical or surgical condition. The clinical conditions most frequently associated with TS are respiratory pathologies, sepsis, neurological disease, endocrine disease, and psychiatric pathologies. The incidence of TS is poorly studied in the critically ill patient setting; furthermore, it is very difficult to determine its incidence, duration, and progression from the current literature. We present the clinical case of a secondary TS in a smoker patient with a history of epilepsy, hospitalized in the ICU for respiratory failure due to viral pneumonia, complicated with bronchospasm, highlighting the diagnostic difficulties in critically ill patients, the presence of multiple trigger factors, and the need to perform an early diagnosis for patient survival.

## Introduction

The term "takotsubo" was introduced by Sato and Dote in 1990, from tako (meaning octopus) and tsubo (meaning a pot). Takotsubo syndrome (TS), also known as ‘‘apical ballooning syndrome’’ or ‘‘stress cardiomyopathy’’, is characterized by regional left ventricular wall motion abnormalities (LVWMA) with a distinct circumferential pattern resulting in a conspicuous ballooning of the left ventricle during systole. The LVWMA may be localized to the apical, mid-apical, mid-ventricular, mid-basal, and basal segments of the left ventricle. The typical TS patient exhibits a unique circumferential left or bi-ventricular contraction abnormality profile that extends beyond a coronary artery supply territory and appears to follow the anatomical cardiac sympathetic innervation [1,2].

The pathological mechanisms of TS remain unclear, and several possible theories have been postulated including catecholamine excess, coronary artery spasm, microvascular dysfunction, and metabolic disturbances.

Recent data indicate that approximately one-third of patients show identifiable physical stressors, including a wide spectrum of medical conditions, such as respiratory disorders [3]. TS is diagnosed in 1-4% of patients who are hospitalized under clinical suspicion of acute coronary syndrome (ACS) [4,5]. Female sex has been identified as a strong risk factor for TS, with a better prognosis than male sex [6].

In 2016, the Heart Failure Association of the European Society of Cardiology introduced the terms primary and secondary TS [7]. In primary TS, acute cardiac symptoms are the primary reason for seeking acute medical care. In secondary TS, the syndrome occurs in patients already hospitalized for a medical or surgical condition. The clinical conditions most frequently associated with TS are respiratory pathologies (acute exacerbation of asthma or COPD, acute pulmonary embolism, pneumonia, asthma), sepsis, neurological disease (subarachnoid hemorrhage, acute head or spinal injury, epileptic seizure), endocrine disease (phaeochromocytoma, thyrotoxicosis), and psychiatric pathologies (acute anxiety, attempted suicide, drug withdrawal syndrome).

In recent years, it has been understood that patient clinical history should also be considered in the diagnostic criteria of TS, and for this reason, the InterTAK criteria were developed. This algorithm is applied to patients presenting to medical emergency departments with chest pain and/or dyspnea. The score facilitates the distinction of stress cardiomyopathy from an ACS, preventing some patients who present without ST-segment elevation on the electrocardiogram from undergoing coronary angiography or other unnecessary invasive diagnostic investigations. The InterTAK Diagnostic score comprises seven parameters (female sex, emotional trigger, physical trigger, absence of ST-segment depression, psychiatric disorders, neurological disorders, and QT prolongation) ranked by their diagnostic importance with a maximum score of 100 points. Depending on the disease prevalence, this means that patients with 30 score points have a predicted probability of <1%, patients with 50% points have a probability of 18%, and patients with a score value > 70 points have a probability of 90% of suffering from TS [8].

TS has a mortality rate of 1-5% of cases, but it is usually reversible within hours to weeks; nevertheless, during the acute stage, a substantial number of patients develop severe complications, such as arrhythmias, heart failure including pulmonary edema and cardiogenic shock, thromboembolism, cardiac arrest, and cardiac rupture [7-9].

Takotsubo in an intensive care unit (ICU) setting is often underdiagnosed, and the wrong treatment could be dangerous for patient survival. Many diseases encountered in the ICU can cause significant stress that potentially triggers TS; indeed, the incidence of TS recorded in patients admitted to intensive care is 4.6% [9]. However, TS is poorly studied in the critically ill patient setting; furthermore, it is very difficult to determine its incidence, duration, and progression from the current literature.

We present the clinical case of a secondary TS in a smoker patient with a history of epilepsy, hospitalized in ICU for respiratory failure due to viral pneumonia, complicated with bronchospasm and TS which lasted that day during ICU stay. Informed consent was obtained for data processing for clinical research purposes. This case report was previously presented as a meeting abstract at the 2024 SMART Italian Congress in Milan on May 27, 2024.

## Case presentation

A 50-year-old woman, an epileptic and a heavy smoker, was admitted to the ICU due to severe bronchospasm that was unresponsive to non-invasive ventilation and intravenous corticosteroids performed in the Emergency Department. The patient had a heart rate of 105 beats per minute, a blood pressure of 125/80 mmHg, and a peripheral saturation of 84% with a respiratory rate of 32 breaths/min upon admission to the hospital. 

**Figure 1:**
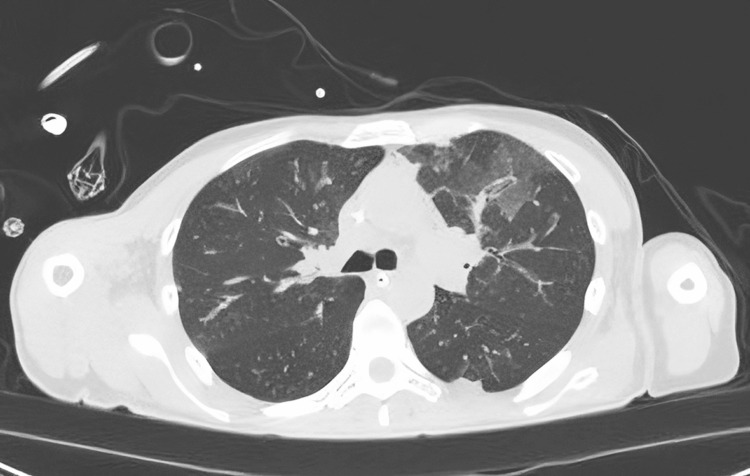
The chest CT showed the presence of multiple areas of ground glass hyperdensity and thickening of the pulmonary interstitium in the consolidative areas, a picture compatible with bilateral interstitial-alveolar pneumonia.

Orotracheal intubation was performed immediately upon admission to the ICU due to severe respiratory failure characterized by hypercapnia and hypoxemia (on blood gas analysis: pH of 7.38, PCO_2_ of 47.1 mmHg, PCO_2_ of 76.9 with FiO_2_ of 60%, and a respiratory rate greater than 40 breaths/min with a high-flow nasal cannula due to the patient's intolerance to non-invasive ventilation). A chest computed tomography (CT) scan revealed the presence of acute interstitial pneumonia (Figure 1). Except for bronchoalveolar lavage (BAL) positive for Rhinovirus, all exams tested were negative for infection.

**Figure 2:**
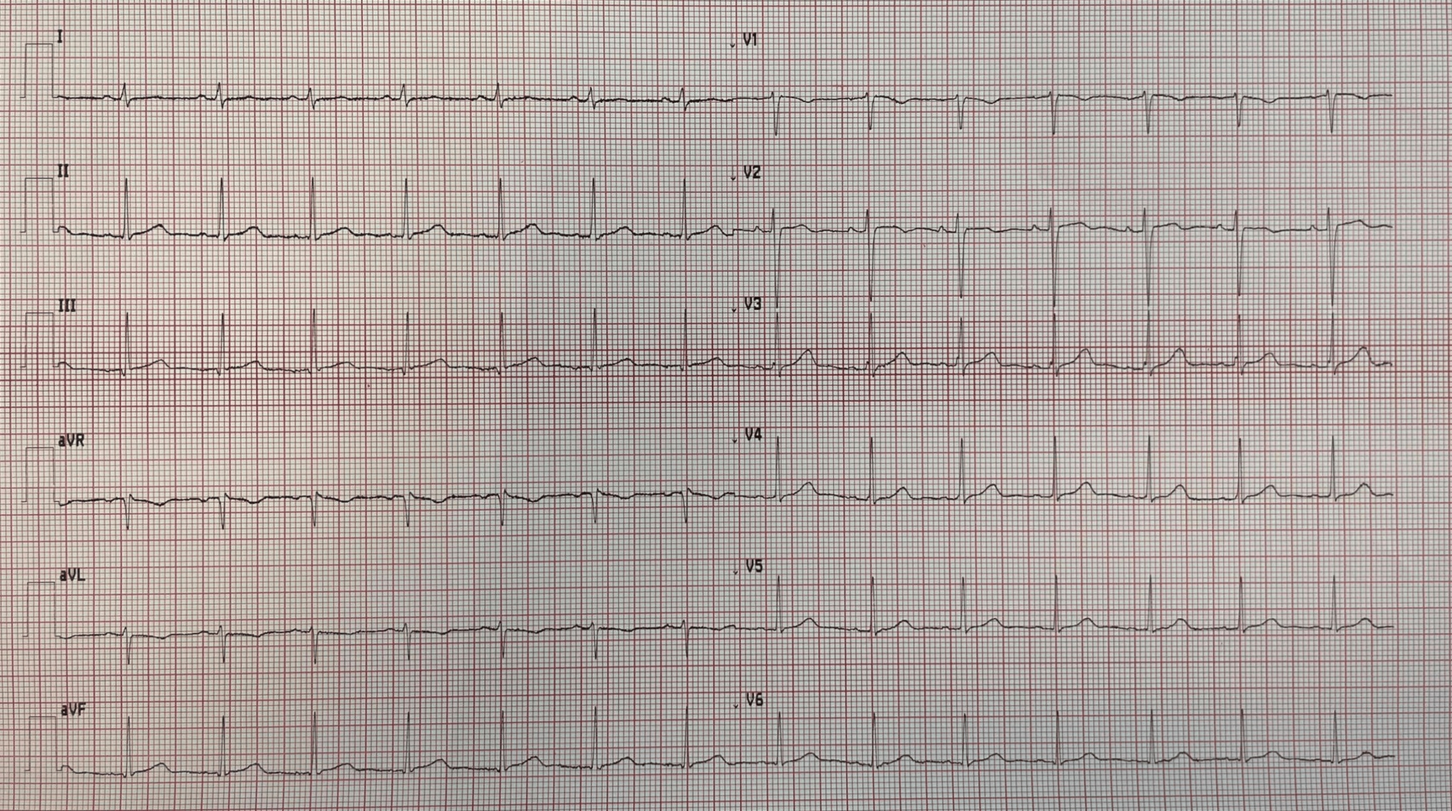
The figure shows the normalization of the electrocardiographic picture contextual to the normalization of the cardiac output and the clinical improvement of the patient.

**Figure 3:**
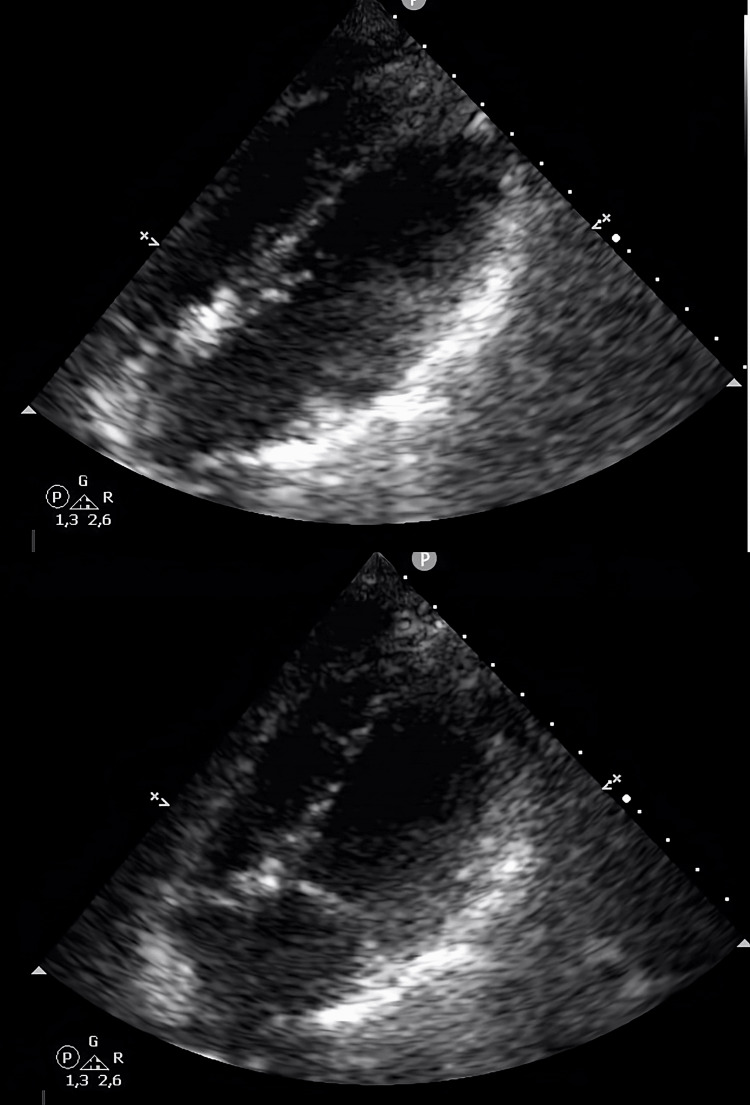
The second control transthoracic echocardiography showed normal ventricular kinetics.

During the acute phase, the patient presented severe bronchospasm and needed deep sedation and neuromuscular blockade for 48 hours. She was treated with methylprednisolone 40 mg, intravenous salbutamol (12 mcg/min), continuous infusion of ketamine (0.5 mg/kg), magnesium, and inhaled corticosteroids. Hemodynamic failure occurred after intubation, and she was treated with norepinephrine (up to the maximum dosage of 0.5 mcg/kg/min). Broad-spectrum antibiotics, including ceftriaxone and clarithromycin, were administered. She had no previous cardiac history, and the initial electrocardiogram was normal. However, on the third day in the ICU, the electrocardiogram showed ST segment elevation in D1, AVL, V3, V4, V5, and V6 leads, compatible with acute anterolateral myocardial infarction (Figure 2). A transthoracic ultrasound by a cardiologist showed a hypokinesis of the mid and apical segments of the left ventricle with an ejection fraction of 40%, highly suspicious for TS (Figure 3). Due to the patient's high clinical instability, the cardiologist did not recommend a coronary angiography study but only recommended clinical monitoring.

**Figure 4:**
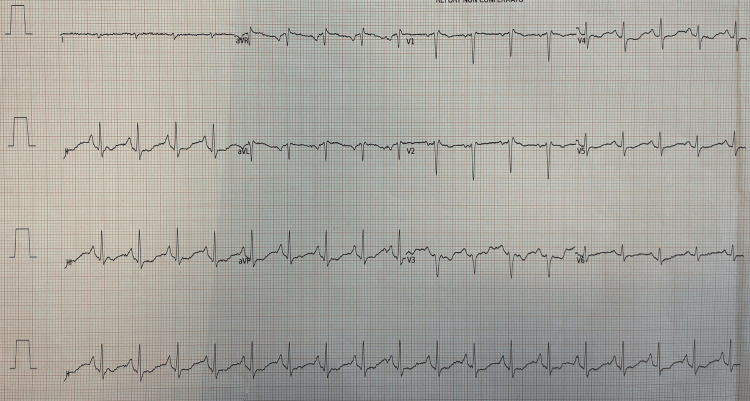
Electrocardiographic abnormalities when the patient showed mid-apical hypokinesia on echocardiography.

**Figure 5:**
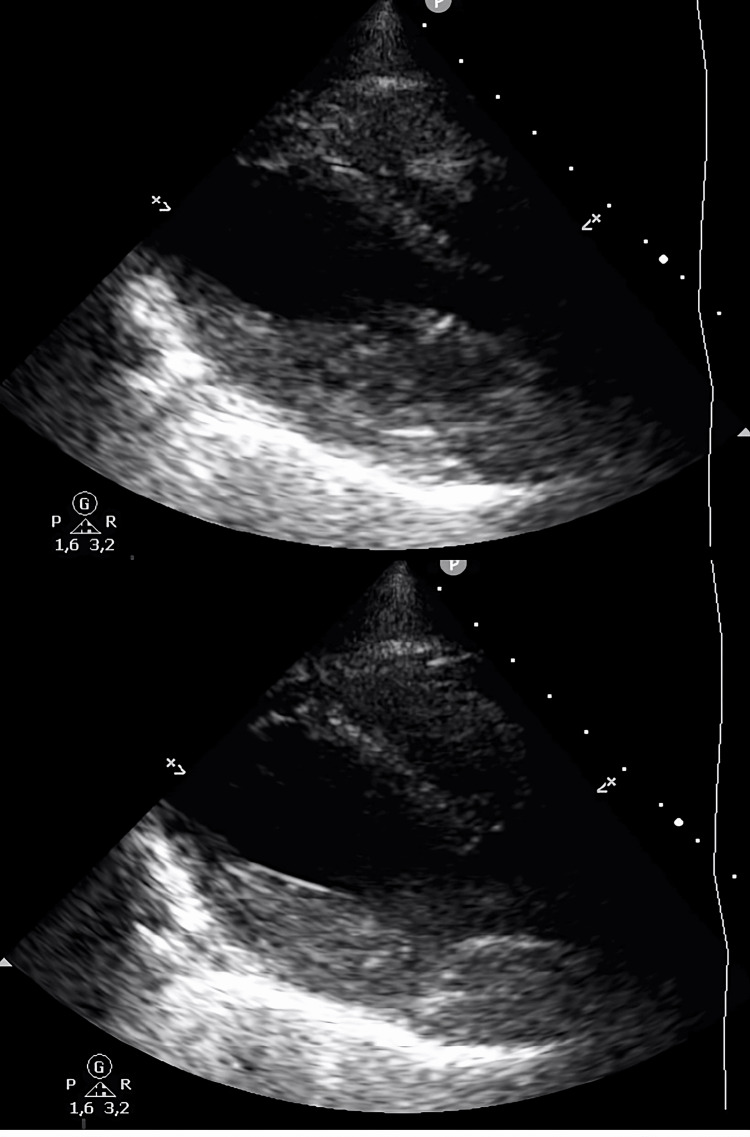
The first transthoracic echocardiography showed a left ventricle with hypokinesia of the mid-apical segments, in particular of the septum, with an ejection fraction of 38-40%.

The InterTAK score of the patient was 83, therefore with a high risk of TS (Table 1). Echocardiography, performed two days after the diagnosis of takotsubo, showed a normal cardiac output with only a septum inter ventricular hypokinesia. The electrocardiographic and ultrasound alterations persisted until the acute phase and the bronchospasm were solved, on day four after admission (Figures 4-5).

**Table 1:** The InterTAK criteria Ref. [8] A value >70 corresponds to a high probability of TS.

Clinical variables	Maximum points	Patient’s points
Female gender	25	25
Emotional stress	24	24
Physical stress	13	13
No ST-segment depression	12	12
Acute former or chronic psychiatric disorder	11	0
Neurological disorder	9	9
Prolonged QTc time	6	0
Total Score	100	83

During hospitalization, the hemodynamics were always unstable, with variable doses of norepinephrine ranging between 0.16 mcg/kg/min and 0.5 mcg/kg/min. The amine support was stopped on the fourth day, with the improvement of the ventricular kinetics, simultaneously with the resolution of the asthmatic condition. On the same day, the patient was successfully extubated, without further cardiac or pulmonary complications.

## Discussion

Takotsubo cardiomyopathy (TCM) is an acute cardiac dysfunction that typically presents hypokinesis of the apical segment of the left ventricle beyond a single coronary artery territory. The recognized pathogenetic mechanisms are sympathetic nervous system over-stimulation, structural and functional alterations in the central nervous systems, catecholamine secretion, alteration in the balance and distribution of adrenergic receptors, impact of hormones including estrogen, epicardial coronary or microvascular spasm, endothelial dysfunction, and genetics as potentially contributing to the cascade of events leading to the onset [10-15].

An increasing number of studies have shown the association of respiratory diseases with TS, including pneumonia and chronic obstructive pulmonary disease (COPD) [16-19], as associated comorbidities lead to increased mortality, increased acute respiratory failure, increased cardiogenic shock, and increased length of hospital days in TS patients [20]. The term “Bronchogenic TS” has been proposed in the literature as a specific form of TS, with an atypical presentation [21].

Our patient was 50 years old, a smoker with epilepsy and asthma, and experienced a respiratory crisis causing hypoxia via ventilation-perfusion mismatch. This triggered compensatory tachypnea, tachycardia, increased work of breathing, and catecholamine surge. COPD and asthmatic patients are given high doses of a beta-agonist combined with ipratropium. As elevated catecholamine levels seem to play a key role in TS, the additional stimulation of cardiac beta-2-adrenergic receptors might potentiate the development of TS under extreme stress, such as hypoxia and acidosis. Acute hypoxia has different organ-system-manifestations and activation of several cardiovascular autonomic processes, and it also exerts a stunning effect on the myocardium, which has been demonstrated in patients with electrocardiogram changes, elevated serum troponin levels, and left ventricular motion abnormalities seen on echocardiogram.

Beyond the diagnostic criteria proposed by the TS Taskforce [7], diagnosing TS in the ICU is challenging. ICU patients very frequently have secondary TS, and the diagnosis of TS is often made due to an unexpected clinical worsening or a difficult weaning from mechanical ventilation or an ECG change. Furthermore, these patients are often sedated and cannot identify their clinical symptoms; performing coronary angiography or other instrumental diagnostic tests may not be feasible due to patients' unstable clinical conditions. Elevated serum troponin levels in these patients may be related to various causes, such as sepsis shock, hypoxia, respiratory failure, intracranial hemorrhage, stroke, and inotrope use, and appear to be an independent predictor of poor prognosis in these patients. ICU patients are usually intubated, and tracheal manipulation can induce a reflex increase in sympathetic activity raising plasma catecholamine levels. The use of beta-agonist bronchodilators can contribute to heart failure; in other patients, administration of exogenous catecholamines (i.e., norepinephrine) appeared to trigger TS [17,22-25]. Vasopressors, inotropes, and mechanical circulatory support are used to treat other forms of cardiogenic shock due to severe left ventricular dysfunction, but these treatments may exacerbate left ventricular outflow obstruction (LVOTO) in the patient with TS and further impair their cardiac output and clinical instability [26].

Given the role that catecholamines and excessive sympathetic stimulation play in TS pathophysiology, the protective role of beta-blocking drugs in the development of TS has been hypothesized; however, their use in traditional dosages does not seem associated with any preventive effect [12].

Early identification of TS in the ICU, as in our patients, can change the management approach. Therefore, some authors propose TS screening with a daily electrocardiogram, so troponin dosage and echocardiography could be proposed in case of electrocardiogram abnormality (or for other obvious clinically justified reasons) [10]. Gupta et al. instead proposed the serial use of echocardiography in the ICU in the early diagnosis and evolutionary monitoring of TS in critically ill patients [27]. Muratsu et al. confirmed that an accurate diagnosis of TCM cannot always be made because cardiac angiography may not be feasible due to the poor general condition of the patient. They defined such cases as clinical TS (cTS) without angiography but meeting all other criteria for TS [28]. In our case report, we made an early diagnosis of TS, considering the multiple pathological triggers, ST elevation, InterTAK criteria value [15], not falling within the classic diagnostic algorithms [7], and consequently highlighting the need for criteria specifically for ICU patients.

In this case, we discovered an association between TS, Rhinovirus pneumonia infection, and acute COPD exacerbation in a 50-year-old woman who was epileptic and hypoxemic and needed fast intubation and admission to the ICU. Suspicions of TS arose when the ECG changed and was confirmed by an echocardiography performed by an expert cardiologist. The patient was extubated once the TS and bronchospasm resolved. While the viruses commonly involved in pneumonia associated with TCM include SARS-CoV2 and influenza type A virus [29,30], there are currently no studies in the literature reporting a connection between TS and Rhinovirus infection. We strongly believe that a deeper understanding of this phenomenon could help clinicians improve the management of these patients and outcomes. It is also crucial to understand the duration of takotsubo. Our patients were successfully extubated after four days also because her takotsubo was resolved.

We believe that the early diagnosis of takotsubo and its lasting could help clinicians improve the management of ICU patients.

## Conclusions

The association of TS with respiratory diseases is still poorly studied in the literature, and even if recognized, it has not been included in the diagnostic elements of the InterTAK criteria. TS remains a potentially life-threatening complication of which the medical community and the intensivist must be aware. Since TS is reversible, it is important to recognize and treat any underlying trigger, especially in patients admitted to the ICU, where the triggers can be multiple (due to pathology, drugs administered, and invasive maneuvers). This case report and literature review may promote a better understanding of TS and have great implications in clinical practice, highlighting the need for further study in critically ill patients.

## References

[1] Y-Hassan S, Tornvall P (2018). Epidemiology, pathogenesis, and management of takotsubo syndrome. Clin Auton Res.

[2] Arbelo E, Protonotarios A, Gimeno JR (2023). 2023 ESC guidelines for the management of cardiomyopathies. Eur Heart J.

[3] Kato K, Cammann VL, Napp LC (2021). Prognostic impact of acute pulmonary triggers in patients with takotsubo syndrome: new insights from the International Takotsubo Registry. ESC Heart Fail.

[4] Akhtar MM, Cammann VL, Templin C, Ghadri JR, Lüscher TF (2023). Takotsubo syndrome: getting closer to its causes. Cardiovasc Res.

[5] Li M, Nguyen CN, Toleva O, Mehta PK (2022). Takotsubo syndrome: a current review of presentation, diagnosis, and management. Maturitas.

[6] Dias A, Núñez Gil IJ, Santoro F (2019). Takotsubo syndrome: state-of-the-art review by an expert panel - part 1. Cardiovasc Revasc Med.

[7] Lyon AR, Bossone E, Schneider B (2016). Current state of knowledge on takotsubo syndrome: a position statement from the taskforce on takotsubo syndrome of the Heart Failure Association of the European Society of Cardiology. Eur J Heart Fail.

[8] Ghadri JR, Wittstein IS, Prasad A (2018). International expert consensus document on takotsubo syndrome (part II): diagnostic workup, outcome, and Management. Eur Heart J.

[9] Templin C, Ghadri JR, Diekmann J (2015). Clinical features and outcomes of takotsubo (stress) cardiomyopathy. N Engl J Med.

[10] Doyen D, Moschietto S, Squara F (2020). Incidence, clinical features and outcome of takotsubo syndrome in the intensive care unit. Arch Cardiovasc Dis.

[11] Li P, Wang Y, Liang J (2022). Takotsubo syndrome and respiratory diseases: a systematic review. Eur Heart J Open.

[12] Sharkey SW, Windenburg DC, Lesser JR (2010). Natural history and expansive clinical profile of stress (tako-tsubo) cardiomyopathy. J Am Coll Cardiol.

[13] Y-Hassan S (2017). Serotonin norepinephrine re-uptake inhibitor (SNRI)-, selective norepinephrine reuptake inhibitor (S-NRI)-, and exogenously administered norepinephrine-induced takotsubo syndrome: analysis of published cases. Int J Cardiol.

[14] Wittstein IS, Thiemann DR, Lima JA (2005). Neurohumoral features of myocardial stunning due to sudden emotional stress. N Engl J Med.

[15] Samul-Jastrzębska J, Roik M, Wretowski D (2021). Evaluation of the interTAK diagnostic score in differentiating takotsubo syndrome from acute coronary syndrome. A single center experience. Cardiol J.

[16] Manfredini R, Fabbian F, Giorgi AD (2014). Heart and lung, a dangerous liaison-tako-tsubo cardiomyopathy and respiratory diseases: a systematic review. World J Cardiol.

[17] Vaz J, Berggren R, Eriksson B (2019). Frequently recurrent takotsubo syndrome in COPD. Case Rep Cardiol.

[18] Khan MA, Howell A, Pham T, Guzman N (2021). Reverse takotsubo cardiomyopathy in the setting of acute asthma exacerbation. Cureus.

[19] Salahuddin FF, Sloane P, Buescher P, Agarunov L, Sreeramoju D (2013). A case of apical ballooning syndrome in a male with status asthmaticus; highlighting the role of B2 agonists in the pathophysiology of a reversible cardiomyopathy. J Community Hosp Intern Med Perspect.

[20] Li P, Lu X, Teng C, Cai P, Kranis M, Dai Q, Wang B (2020). The impact of COPD on in-hospital outcomes in patients with takotsubo cardiomyopathy. Int J Chron Obstruct Pulmon Dis.

[21] Madias JE (2015). Bronchogenic stress cardiomyopathy’, a subset of takotsubo syndrome. Cardiology.

[22] Franco E, Dias A, Figueredo VM, Hebert K (2014). Is acute respiratory failure requiring mechanical ventilation associated with development of takotsubo cardiomyopathy in the critical care setting?. Int J Cardiol.

[23] Abbasi D, Faiek S, Siddiqui WJ, Lopez-Candales A (2022). Assessing the role of high-dose β-agonists use in triggering takotsubo syndrome during asthma exacerbation. Perm J.

[24] Khwaja YH, Tai JM (2016). Takotsubo cardiomyopathy with use of salbutamol nebulisation and aminophylline infusion in a patient with acute asthma exacerbation. BMJ Case Rep.

[25] Mendoza I, Novaro GM (2012). Repeat recurrence of takotsubo cardiomyopathy related to inhaled beta-2-adrenoceptor agonists. World J Cardiol.

[26] Ahmed T, Lodhi SH, Haigh PJ, Sorrell VL (2024). The many faces of takotsubo syndrome: a review. Curr Probl Cardiol.

[27] Gupta P, Chockalingam A (2019). Characterising the clinical spectrum, diagnosis and outcomes in secondary stress cardiomyopathy. Heart Int.

[28] Muratsu A, Muroya T, Kuwagata Y (2019). Takotsubo cardiomyopathy in the intensive care unit. Acute Med Surg.

[29] Li P, Shi A, Lu X (2024). Incidence and impact of takotsubo syndrome in hospitalized patients with COVID-19. Tex Heart Inst J.

[30] Kanelidis AJ, Miller PJ, Singh A, Addetia K, Lang RM (2022). Takotsubo syndrome from coronavirus disease 2019. J Am Soc Echocardiogr.

